# Allogeneic HSCT for Autoimmune Diseases: A Retrospective Study From the EBMT ADWP, IEWP, and PDWP Working Parties

**DOI:** 10.3389/fimmu.2019.01570

**Published:** 2019-07-04

**Authors:** Raffaella Greco, Myriam Labopin, Manuela Badoglio, Paul Veys, Juliana M. Furtado Silva, Mario Abinun, Francesca Gualandi, Martin Bornhauser, Fabio Ciceri, Riccardo Saccardi, Arjan Lankester, Tobias Alexander, Andrew R. Gennery, Peter Bader, Dominique Farge, John A. Snowden

**Affiliations:** ^1^Unit of Hematology and Bone Marrow Transplantation, IRCCS San Raffaele Scientific Institute, Vita-Salute San Raffaele University, Milan, Italy; ^2^EBMT Paris Study Office/CEREST-TC - Department of Haematology, Saint Antoine Hospital - INSERM UMR 938 - Université Pierre et Marie Curie, Paris, France; ^3^Department of Bone Marrow Transplantation, Great Ormond Street Hospital, London, United Kingdom; ^4^Great North Childrens' Hospital, Institute of Cellular Medicine, Newcastle University, Newcastle upon Tyne, United Kingdom; ^5^Divisione Ematologia e Trapianto di Midollo, IRCCS AOU San Martino-IST, Genova, Italy; ^6^Medizinische Klinik und Poliklinik I, Carl Gustav Carus University Hospital, Dresden, Germany; ^7^Department of Haematology, Careggi University Hospital, Florence, Italy; ^8^IEWP-EBMT Chair: Department of Pediatrics, University Medical Centre, Leiden, Netherlands; ^9^Department of Rheumatology and Clinical Immunology, Charité-University Medicine Berlin, Berlin, Germany; ^10^PDWP-EBMT Chair; Division for SCT and Immunology, Department for Children and Adolescents, Frankfurt, Germany; ^11^Unité de Médecine Interne: Maladies Auto-immunes et Pathologie Vasculaire (UF 04), Hôpital St-Louis, AP-HP, Paris, France; ^12^Centre de Référence des Maladies Auto-Immunes Systémiques Rares d'Ile-de-France, Filière FAI2R, Paris, France; ^13^EA 3518, Université Denis Diderot, Paris, France; ^14^Department of Internal Medicine, McGill University, Montreal, QC, Canada; ^15^ADWP-EBMT Chair; Department of Haematology, Sheffield Teaching Hospitals NHS Foundation Trust, Sheffield, United Kingdom

**Keywords:** allogeneic hematopoietic stem cell transplantation, autoimmune diseases, long-term outcome, hematological autoimmune diseases, non-hematological autoimmune diseases

## Abstract

**Background:** This retrospective study assessed the use and long-term outcome of allogeneic hematopoietic stem cell transplantation (HSCT) in patients with severe autoimmune diseases (ADs), reported to the European Society for Blood and Marrow Transplantation (EBMT) registry.

**Methods:** Between 1997 and 2014, 128 patients received allogeneic HSCT for various hematological (*n* = 49) and non-hematological (*n* = 79) refractory ADs. The median age was 12.7 years (0.2–62.2). Donors were syngeneic for seven, matched related for 46, unrelated for 51, haploidentical for 15, and cord blood for nine patients.

**Results:** The incidence of grades II-IV acute graft-vs.-host disease (GvHD) was 20.8% at 100 days. Cumulative incidence of chronic GvHD was 27.8% at 5-years. Non-relapse mortality (NRM) was 12.7% at 100-days. Overall survival (OS) and Progression-Free Survival (PFS) were 70.2 and 59.4% at 5-years, respectively. By multivariate analysis, age <18 years, males, and more recent year of transplant were found to be significantly associated with improved PFS. Reduced conditioning intensity was associated with a lower NRM. On a subgroup of 64 patients with detailed information a complete clinical response was obtained in 67% of patients at 1-year.

**Conclusions:** This large EBMT survey suggests the potential of allogeneic HSCT to induce long-term disease control in a large proportion of refractory ADs, with acceptable toxicities and NRM, especially in younger patients.

## Background

Allogeneic hematopoietic stem cell transplantation (HSCT) is a potentially curative standard treatment for a variety of malignant and non-malignant disorders (1). During the last decade major changes have occurred in the field of HSCT (1–5). Limitations for the transplant procedures have been modified because of the introduction of less aggressive conditioning regimens, improved patient selection, better supportive care, and recent advances in graft-vs.-host disease (GvHD) prophylaxis, thus representing an intriguing approach for patients affected by non-malignant disorders and autoimmune diseases (ADs) (6–16).

Several studies have investigated the potential application of autologous and allogeneic HSCT to control otherwise resistant disease activity in severe ADs (9, 17, 18). In particular, the rationale for autologous HSCT in ADs has been to ablate and reset the autoreactive immune system (9, 18–20). However, some patients may develop disease flares resulting either from incomplete eradication of autoreactive memory cells or re-induction of autoimmunity driven by genetic predisposition (9, 20).

For patients with refractory ADs, allogeneic HSCT offers a more potent eradication of autoreactive cells, achieved through the conditioning regimen, combined with the replacement by a new donor immune system, with the potential to develop tolerance to both allo- and auto-antigens (21–23). Nevertheless, allogeneic HSCT has been a rare treatment option for ADs compared with autologous HSCT (18, 24–31). The area where allogeneic HSCT has mostly been used has been immune cytopenia (27). Some patients clearly benefited from this treatment (31), despite considerable toxicity and non-relapse mortality (NRM) reported in the past (9, 18). The rationale for the use of allogeneic HSCT has gained additional traction more recently, as monogenic defects associated with autoimmunity and immunodeficiency have been identified (32–37).

The European Society for Blood and Marrow Transplantation (EBMT) Autoimmune Diseases (ADWP), Inborn Errors (IEWP) and Pediatric (PDWP) Working Parties conducted a retrospective survey to analyze long-term disease outcome and toxicity of allogeneic HSCT in refractory ADs patients.

## Methods

### Study Design

This study is a retrospective multicenter study analyzing EBMT registry data. Additional information was collected using a specific questionnaire. The study was approved by the ADWP and participating EBMT centers reported data from patients affected by ADs (hematological and non-hematological ADs), including those with autoinflammatory and genetic components, who had been treated with allogeneic HSCT from the first registration in 1997 to the end of 2014. Patients with a previous allogeneic HSCT for a conventional hematological indication have been included. Patients transplanted for a malignant hematological disease with a concomitant AD were excluded from the analysis. All EBMT teams are required to obtain signed informed consent for transfer of anonymized data via standardized minimal essential data forms in accordance with data protection regulations.

The following variables were analyzed from the EBMT database: patient gender, patient age at transplantation, disease classification at time of diagnosis according to primary disease established criteria, conditioning regimen, graft characteristics, incidence of acute, and chronic GvHD.

In addition, long-term side effects (secondary autoimmune disease or malignancy, viral reactivations), previous and post-transplant treatments, and disease status at last follow-up were analyzed in the subgroup of 64 patients with completed questionnaires. Disease characteristics (severity, refractoriness to standard treatments, revision and reclassification of all diagnoses in view of the spectrum of pediatric ADs, including those with autoinflammatory and overlap with other genetic diseases and inborn errors) were assessed by local physician.

### Definitions

All outcomes were measured from the time of stem cell infusion.

Engraftment was defined as a persistent blood cells count above a predefined level (neutrophils >0.5 × 10^9^/l per three consecutive days and platelets >20 × 10^9^/l, unsupported by transfusions, for 3 days), starting from the day of transplantation. Progression-free survival (PFS) was defined as survival without evidence of disease reactivation.

The response to transplant for immune cytopenia was defined according to the following criteria: *complete remission* (CR) for the normalization of blood counts with hemoglobin >12 g/dL, neutrophils >1.5 × 10^9^/L and platelets >150 × 10^9^/L; *partial remission* (PR*)* for hemoglobin >8 and ≤12 g /dl, neutrophils >0.5 × 10^9^/L, and ≤1.5 × 10^9^/L and platelets >50 × 10^9^/L and ≤150 × 10^9^/L; *active disease* for level ≤8 g /dl, neutrophils ≤0.5 × 10^9^/L and platelets ≤50 × 10^9^/L) (30). For AD other than cytopenias response was assessed by local physician based on the following classification: *complete remission* in the absence of all clinical and laboratory autoimmune disease activity); *partial remission* for low disease activity with persistence of minor disease specific symptoms; *active disease* in case of no response or response not fulfilling CR/PR criteria. Failure to respond was assessed according to the above criteria by the local physician in charge of the AD based on an inability to achieve a PR or CR at any time point. Major relapse was assessed according to the above criteria by the local physician in charge of the AD based on progression from a CR or PR to active disease. Minor relapse was assessed according to the above criteria by the local physician in charge of the AD based on progression from a CR to a PR. These criteria applied irrespective of the AD (i.e., include immune cytopenias).

Overall survival (OS) was defined as the time from allogeneic HSCT to death, regardless of the cause. NRM was defined as death without evidence of relapse or progression.

### Statistics

The probabilities of OS and progression-free survival (PFS) were calculated by using the Kaplan-Meier method. Cumulative incidence was used to estimate the following endpoints: NRM, relapse incidence (RI), acute and chronic GvHD, to accommodate for competing risks. To study acute and chronic GvHD, we considered relapse and death to be competing events.

Multivariate analysis included all variable associated with one endpoint in univariate analysis: AD diagnosis (hematological vs. non-hematological diseases), sex, age at HSCT (cut-off 18 years), donor type (matched related donor vs. others), year of HSCT (before or after 2009), conditioning regimen (myeloablative vs. reduced-intensity conditioning) and GvHD prophylaxis (*ex-vivo* or *in-vivo* T-cell depletion). Results were expressed as the hazard ratio (HR) with the 95% confidence interval (95% CI). Statistical analyses were performed with SPSS 24.0 (SPSS Inc., Chicago, IL, USA) and R 3.4.0 (38).

## Results

### Overall Population and Main Transplant Outcomes

#### Patients and HSCT Characteristics

Between 1997–2014 inclusive, 128 patients received allogeneic HSCT for various hematological (*n* = 49) and non-hematological (*n* = 79) refractory AD. Disease and transplant characteristics are summarized in Tables 1, 2. Patients were treated at 63 centers in 21 countries (Table 3).

**Table 1:** Disease classification (overall population; *n* = 128).

**Overall population (*n* = 128)**	***N* (%)**
**Non hematological disorders**, ***n*** **=** **79 (61.7%)**	
MS	4 (3.1%)
SLE	5 (3.9%)
Other CTD	2 (1.6%)
Wegener	1 (0.8%)
Behcet	2 (1.6%)
Other vasculitis	5 (3.9%)
RA	2 (1.6%)
JIA	13 (10.2%)
CIDP	1 (0.8%)
NMO	5 (3.9%)
Other neurological disorders	2 (1.6%)
Crohn's disease	7 (5.5%)
Other IBD	13 (10.2%)
Other Ads	17 (13.3%)
**Hematological disorders**, ***n*** **=** **49 (38.3%)**	
ITP	6 (4.7%)
AIHA	11 (8.6%)
Evans syndrome	15 (1.8%)
Other non-malignant hematological disorders	17 (13.3%)

*The table includes details about disease characteristics of all the patients, divided as “hematological disorders” and “non-hematological disorders.” MS, multiple sclerosis; SLE, systemic lupus erythematosus; RA, rheumatoid arthritis; JIA, juvenile idiopathic arthritis; CIDP, chronic inflammatory demyelinating polyneuropathy; NMO, neuromyelitis optica; ITP, Immune thrombocytopenia; AIHA, autoimmune hemolytic anemia; IBD, inflammatory bowel disease; ADs, autoimmune diseases; CTD, connective tissue disease*.

**Table 2:** Transplant details (overall population; *n* = 128).

**Variables**	**Number of patients**	**%**
**Donor**		
MRD	46	35.94
UD	51	39.84
MMRD	15	11.72
Syngeneic	7	5.47
Cord blood	9	7.03
**Stem Cell Source**		
Peripheral blood	67	52.34
Bone marrow	52	40.62
Cord blood	9	7.03
**Conditioning regimen**		
RIC	48	39.34
MAC	74	60.66
Missing information	6	
***Ex-vivo*** **manipulation**		
No	114	89.06
Yes	14	11.67
***In vivo*** **TCD**		
No	32	26.89
Yes:	87	73.11
ATG	41	
Alemtuzumab	46	
Missing	9	
**Post-transplant GvHD prophylaxis**	115	89.84%
CSA	21	
CSA+MTX	31	
CSA+ MMF	47	
Tacrolimus + MMF	2	
Tacrolimus + MTX	4	
PT-Cy	2	
Other	8	
Missing information	8	

*The table includes details about the graft and the conditioning regimen for all the patients. MRD, Matched related donor; UD, unrelated donor; MMRD, Mismatched related donor; RIC, reduced-intensity; MAC, myeloablative; TCD, T-cell depletion; ATG, anti-thymocyte globulin; CSA, cyclosporine; MMF, mycophenolate mofetil; MTX, methotrexate; PT-Cy, post-transplant cyclophosphamide*.

**Table 3:** Center distribution by country (overall population; *n* = 128).

**Center**	**Number of patients**	**%**
United Kingdom	42	33
Germany	16	13
Italy	13	10
Netherlands	10	8
Spain	5	4
Russia	2	2
Belgium	2	2
France	6	5
Switzerland	2	2
Turkey	9	7
Hungary	3	2
Czech Republic	2	2
Israel	2	2
Austria	4	3
Sweden	4	3
Norway	1	1
Portugal	1	1
Belarus	1	1
Lithuania	1	1
Poland	1	1
Lebanon	1	1
Total	128	100%

*Details of the 63 participating centers from 21 European countries. The predominant country of activity was United Kingdom, who made up 33% of the activity*.

The median age of patients at HSCT was 12.7 years (range 0.2–62.2). Eighteen patients received a previous transplant, mainly autologous HSCT (*n* = 15). Median time from diagnosis to transplant was 3.8 years (range 0.01–27.2). Median follow-up was 49 months (range 21–87).

Donors were matched related donors (MRD) for 46 patients, unrelated donors (UD) for 51, mismatched related donor (MMRD) for 15, unrelated cord blood for nine, and syngeneic for seven patients.

The graft source was peripheral blood stem cells (PBSCs) in 67, bone marrow (BM) in 52, and cord blood in nine patients.

Conditioning was classified (39) as myeloablative (MAC) in 74 and reduced-intensity (RIC) in 48 patients. Total body irradiation (TBI) was included in the conditioning regimen for 15 patients. Busulfan-based regimen was used in 35 patients, while 24 patients received a treosulfan-based regimen.

*Ex-vivo* T-cell depletion (TCD) was performed in 14 cases. Serotherapy with anti-thymocyte globulin (ATG) was given in 41 patients and with alemtuzumab in 46 patients. Post-transplant GvHD prophylaxis was given in 115 patients, mainly based on cyclosporine, associated either with methotrexate (27%) or mycophenolate mofetil (41%).

#### Engraftment and Graft Rejection

All except seven patients experienced sustained donor cell engraftment.

Engraftment (see Methods) was obtained in all patients within a median of 14.5 days from HSCT, both for neutrophils and platelets. Graft failure was reported in seven patients, thus resulting in an overall primary graft failure rate of 5.5%.

#### GvHD

In all, 53 out of 128 patients developed acute GvHD, and the incidence of grades II-IV acute GvHD was 20.8% (95% CI: 14.1–28.5) at 100-days. Nine patients reported grades III-IV acute GvHD. Cumulative incidence of chronic GvHD was 27.8% (95% CI: 19.3–36.9) at 5-years.

#### Survival and Risk Factors for Outcome

**Figure 1:**
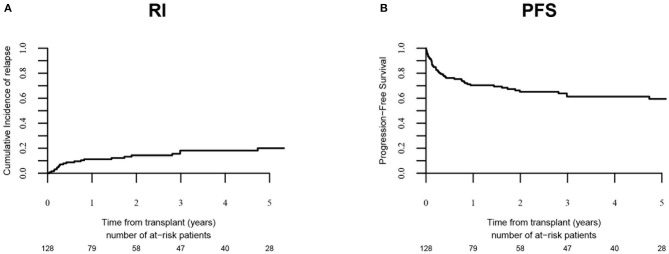
Relapse incidence (RI) and progression-free survival (PFS). RI and PFS in overall population (*n* = 128) are shown in **(A,B)** respectively.

**Figure 2:**
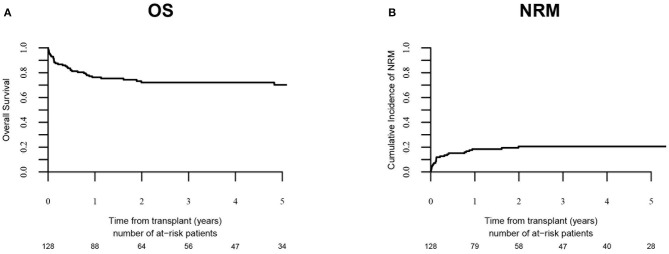
OS and cumulative incidence of NRM. OS and cumulative incidence of NRM in overall population (*n* = 128) are shown in **(A,B)**, respectively.

Relapse incidence (Figure 1) was 18.1 % (95% CI: 11.4–26.1) at 3-years and 20% (95% CI: 12.6–28.7) at 5-years. PFS was 59.4% at 5-years (95% CI: 49.9–69). NRM (Figure 2) was 12.7% (95% CI: 7.6–19.2) at 100-days, and 20.5% at 5-years (95% CI: 13.8–28.2). The OS was 70.2% (95% CI: 61.5–78.9) at 5-years.

Significant associations were observed by univariate analysis. Higher NRM (28.7 vs. 12.3%; *p* = 0.02) and lower PFS (49 vs. 73.3%; *p* = 0.02) were registered in females. Adult patients presented higher RI (31.2% vs. 10.3%; *p* = 0.002), lower PFS (46.8 vs. 69.8%; *p* = 0.009) and higher rates of acute GvHD (42.2 vs. 21.3%; *p* = 0.005) as compared with children. A more recent year of transplant was associated with higher PFS (71.6 vs. 50.4%; *p* = 0.008) and lower rates of acute GvHD (16.3 vs. 38.8%; *p* = 0.007). Non-hematological disorders showed a trend toward a better OS (77.9 vs. 62.9%; *p* = 0.05). Lower rates of chronic GvHD were reported in HSCT from MRD (11.7 vs. 26%; *p* = 0.04). No significant association was reported for ATG or alemtuzumab.

A multivariate analysis (Table 4) of prognostic factors was performed. Age more than 18 years was associated with a higher RI (HR: 3.72; 95% CI: 1.38–10; *p* = 0.009), a higher incidence of chronic GvHD (HR: 3.75; 95% CI: 1.67–8.39; *p* = 0.001) and a lower PFS (HR: 2.07; *p* = 0.02). A more recent year of transplant was associated with a lower NRM (HR: 0.86; *p* = 0.003 and chronic GvHD (HR: 0.88; *p* = 0.009); a better PFS (HR: 0.9; *p* = 0.01) and OS (HR: 0.89; *p* = 0.003). Females were associated with a higher NRM (HR: 3.94; *p* = 0.008) and lower PFS (HR: 2.5; *p* = 0.009) and OS (HR: 2.61; *p* = 0.02). RIC regimen was associated with a lower NRM (HR: 0.31; *p* = 0.02). MRD was associated with a lower incidence of acute GvHD (HR = 0.24, 95% CI: 0.08–0.75; *p* = 0.01). There was no significant association with diagnosis and centers.

**Table 4:** Multivariate analysis (overall population; *n* = 128).

	**Relapse/progression**		**NRM**		**PFS**		**OS**		**Acute GVHD II-IV**		**Chronic GVHD**	
	**HR (95% CI)**	***p*-value**	**HR (95% CI)**	***p*-value**	**HR (95% CI)**	***p*-value**	**HR (95% CI)**	***p*-value**	**HR (95% CI)**	***p*-value**	**HR (95% CI)**	***p*-value**
Female vs. male	1.72 (0.65–4.56)	0.278	3.94 (1.44–10.8)	**0.0075**	2.5 (1.26–4.96)	**0.00876**	2.61 (1.2–5.67)	**0.0152**	1.59 (0.69–3.69)	0.28	0.67 (0.3–1.5)	0.329
Adults vs. children	3.72 (1.38–10)	**0.0092**	1.09 (0.45–2.66)	0.852	2.07 (1.1–3.88)	**0.0232**	1.24 (0.6–2.56)	0.569	0.968 (0.4–2.32)	0.942	3.75 (1.67–8.39)	**0.00133**
Year of transplant	0.973 (0.86–1.1)	0.653	0.858 (0.78–0.948)	**0.00248**	0.908 (0.84–0.98)	**0.013**	0.885 (0.82–0.959)	**0.00293**	0.943 (0.85–1.04)	0.243	0.875 (0.79–0.97)	**0.0087**
Immune cytopenia vs. other	2.05 (0.75–5.63)	0.163	0.865 (0.35–2.16)	0.756	1.18 (0.61–2.28)	0.617	1.62 (0.78–3.35)	0.193	1.41 (0.62–3.24)	0.414	0.698 (0.29–1.69)	0.427
MRD vs. other donors	1.79 (0.66–4.86)	0.255	0.56 (0.22–1.46)	0.235	0.949 (0.48–1.86)	0.879	0.819 (0.39–1.74)	0.602	0.244 (0.08–0.746)	**0.0133**	0.603 (0.24–1.53)	0.286
RIC vs. MAC	1.69 (0.63–4.53)	0.294	0.306 (0.12–0.814)	**0.0176**	0.69 (0.36–1.31)	0.258	0.648 (0.31–1.34)	0.243	0.87 (0.38–2.01)	0.744	0.998 (0.46–2.19)	0.995
Centers	NA (0.65–4.56)	0.952	NA (1.44–10.8)	0.953	NA (1.26–4.96)	0.943	NA (1.2–5.67)	0.945	NA (0.69–3.69)	0.948	NA (0.3–1.5)	0.948

*The table showed details of the multivariate analysis based on AD diagnosis (hematological vs. non-hematological diseases), sex, age at HSCT (cut-off for adults at 18 years), donor type (matched related donor vs. others), year of HSCT (before or after 2009), conditioning regimen (myeloablative vs. reduced-intensity conditioning) and centers. MAC, myeloablative conditioning regimen; RIC, reduced-intensity conditioning regimen; MRD, matched related donor. Bold values are intended as statistically significant*.

### Subgroup of 64 Patients With Additional Information

Among the total 128 patients who received allogeneic HSCT between 1997 and 2014, we received detailed questionnaires on long-term outcomes from 64 patients. These patients were treated at 24 centers in 13 countries (United Kingdom, Italy, Germany, Spain, Russia, Belgium, France, Switzerland, Turkey, Hungary, Lebanon, Canada and Czech Republic).

Disease characteristics for this cohort of patients are shown in Table 5; in view of the spectrum of pediatric ADs, including those with autoinflammatory and overlap with other genetic diseases and inborn errors, all diagnoses were reviewed and classified accordingly.

**Table 5:** Reviewed disease classification (selected cohort of patients; *n* = 64).

**Selected population (*n* = 64)**	***N* (%)**
**Non-hematological disorders**, ***n*** **=** **43 (67.2%)**	
SLE	3 (4.7%)
Cq1 deficiency (SLE)	1 (1.6%)
GPA /Wegener's granulomatosis	1 (1.6%)
Behcet's disease	1 (1.6%)
RA	1 (1.6%)
JIA	7 (10.9%)
NMO	4 (6.3%)
Crohn's disease	5 (7.8%)
Other IBD	4 (6.3%)
IPEX syndrome	1 (1.6%)
Multi-systemic inflammatory disease syndrome	3 (4.7)%
Lichen myxoedematosus	1 (1.6%)
MKD/TRAPS polymorphism	1 (1.6%)
Glanzmann's Thrombasthenia	1 (1.6%)
Njimegen Breakage Syndrome	1 (1.6%)
Tricohepatoenteric syndrome	1 (1.6%)
Other Ads	7 (10.9%)
**Hematological disorders**, ***n*** **=** **21 (32.8%)**	
ITP	2 (3.1%)
AIHA	7 (10.9%)
Evans syndrome	9 (14.1%)
Severe B-, T-, NK-cell immunodeficiency	1 (1.6%)
SCID	1 (1.6%)
PWCA	1 (1.6%)

*The table includes details about disease characteristics of the selected cohort of patients with more detailed characteristics (n = 64), divided as “hematological disorders” and “non-hematological disorders.” In view of the spectrum of pediatric ADs, all diagnoses were reviewed and classified according to the study questionnaire. AIHA, autoimmune hemolytic anemia; ITP, immune thrombocytopenia; JIA, juvenile idiopathic arthritis; SCID, severe combined immuno deficiency; IPEX, immune dysregulation, polyendocrinopathy, enteropathy, X-linked; GPA, granulomatosis with polyangiitis; NMO, neuromyelitis optica; RA, rheumatoid arthritis; PWCA, pure white cell aplasia; TRAPS, TNF receptor-associated periodic syndrome; IBD, inflammatory bowel disease; SLE, systemic lupus erythematosus; ADs, autoimmune diseases; MKD, mevalonic kinase deficiency*.

The diagnosis of AD was hematological (*n* = 21) and non-hematological (*n* = 43), among pediatric (*n* = 45) and adult (*n* = 19) populations. The median age of patients at HSCT was 11.14 years (range 1.22–51.57).

All patients were refractory to previous immunosuppressive therapies (median of four lines of treatments, range 1–13), including cyclophosphamide (*n* = 16), biological therapies (*n* = 41, including rituximab in 20 patients and infliximab in 14), cytotoxic drugs (*n* = 30), oral immunosuppression (*n* = 37), intravenous immunosuppression (*n* = 22), and others (*n* = 29). Median time from diagnosis to transplant was 5 months (range 0.3–27).

Eight patients (12%) had received a previous autologous transplant. Two patients received an allogeneic HSCT for a conventional hematological indication in the past (Non-Hodgkin Lymphoma and Severe Aplastic Anemia).

The graft source was PBSCs in 38, BM in 24, and cord blood in two patients. Donor sources were as follows; 41% MRD (*n* = 26), 42% UD (*n* = 27), 9% MMRD (*n* = 6), 5% syngeneic (*n* = 3), and 3% cord blood (*n* = 2). Median CD34+ and CD3+ cell doses infused were 6.52 × 10^6^/Kg and 192.1 × 10^6^/Kg, respectively.

Conditioning was MAC in 35 and RIC in 29 patients. A fludarabine-based regimen was used in 50 (80%) patients, in association with treosulfan for 17 patients, melphalan for 14 patients, and cyclophosphamide for 11 patients. Nine patients received TBI. *Ex-vivo* TCD was performed in seven cases. Serotherapy with ATG was given in 16 patients, while 30 patients received alemtuzumab. Post-transplant GvHD prophylaxis was cyclosporine-based for the majority of patients (*n* = 54), with the addition of mycophenolate mofetil (MMF) in 33 cases; four patients received tacrolimus, and two patients were treated with sirolimus.

#### Treatment-Related Complications

##### GvHD

The incidence of grades II-IV acute GvHD was 16.4% (95% CI: 8.4–26.8) at 100-days; severe acute GvHD was reported only in four patients. Cumulative incidence of chronic GvHD was 32.5% (95% CI: 20.8–44.8) at 5-years; extensive manifestations of chronic GvHD were reported in 11 patients. In univariate analysis the incidence of chronic GvHD was significantly lower in patients receiving *in-vivo* TCD (24.3 vs. 52.9%; *p* = 0.02).

##### Secondary autoimmune diseases

Seven patients (11.3%) developed secondary autoimmune diseases, distinct from their primary disease, at a median time of 15 months (7.9–43.8), including autoimmune thrombocytopenia (*n* = 1), autoimmune thyroid diseases (*n* = 3), psoriasis (*n* = 1), autoimmune hemolytic anemia (AIHA *n* = 1), Guillain-Barré syndrome (*n* = 1), and other secondary AD not otherwise specified (*n* = 1).

##### New malignancies

One case of new malignancy (lymphoma) was reported at 62 months post-transplant.

##### Infections

Viral reactivations were reported in a total of 33 patients, including Cytomegalovirus (CMV; *n* = 14), Epstein-Barr Virus (EBV; *n* = 10), Adenovirus (*n* = 5), polyoma BK virus (*n* = 3; hemorrhagic cystitis in two cases), Herpes Simplex Virus (HSV, *n* = 2), Human Herpesvirus 6 (HHV6; *n* = 3), and Varicella-Zoster Virus (VZV; *n* = 2). Seven cases of invasive fungal infection were reported (on which one aspergillosis and three candidiasis). Ten bacterial infections (only four patients developed infection from Gram-negative bacteria) were observed and five patients developed pneumonia. Renal failure was reported in four cases. Other adverse events were reported in the long-term follow-up of these patients: severe post-transplant lymphopenia (*n* = 1), severe osteopenia (*n* = 2; possibly due to prolonged previous treatment with steroids), renal plus liver impairment (*n* = 1), cerebral hemorrhage (*n* = 1), chronic lung disease (*n* = 1), multi-organ failure (MOF; *n* = 2).

#### Long-Term Response to Treatment

At the time of transplantation, all patients had active disease refractory to standard therapy. Median follow-up was 67 months (IQR 36.5–104 months). At 100 days post-HSCT, response was achieved in 79% of patients (complete remission in 39 patients, partial remission in 10 patients). At the last follow-up complete clinical response of refractory AD was obtained in 67.2% of patients (*n* = 43), while partial remission was reported in 6.3% (*n* = 4).

Incidence of relapse/progression was 20.3% (95% CI: 10.6–32.2) at 5 years, with no differences between immune cytopenia (16.2%) and other ADs (20.6%). Post-HSCT autoimmune disease specific treatment was required for 12 patients, mainly based on steroids (*n* = 7), in combination with rituximab (*n* = 2), intravenous immunoglobulins (*n* = 1), bortezomib (*n* = 1), MMF (*n* = 2), cyclophosphamide (*n* = 1). Adalimumab (*n* = 1), plasmapheresis (*n* = 1) or second allogeneic HSCT (*n* = 1) were also reported.

At last follow-up 39 patients are progression and immunosuppression-free survivors.

#### Long-Term Outcomes

Results confirmed those found in the entire population. OS was 76% at 5-year (95% CI: 64.8–87.3). The primary cause of death was divided between disease (progression of AD; *n* = 6), NRM (*n* = 9; seven patients succumbed to infections, and two with MOF). All causes of death classified by local physician as NRM, were related to drug toxicity and transplant; none of these patients experienced disease recurrence or received AD specific treatment after HSCT. Cumulative incidence of NRM was 14.9% at 5-year (95% CI: 7.3–25.1) and PFS was 64.8% at 5-year (95% CI: 52.1–77.6).

By univariate analysis, there was no significant differences between immune cytopenia and other ADs in terms of RI (16.2 vs. 18.6%, respectively, *p* = 0.91), OS (76.2 vs. 79.9%), respectively, *p* = 0.59) and PFS (73.8 vs. 64.3%, *p* = 0.63). A more recent year of transplant, after 2009, was significantly associated with improved outcomes: higher PFS (76.3 vs. 55.6%; *p* = 0.03), higher OS (90.7 vs. 64.3%; *p* = 0.005), and lower rates of chronic GvHD (19 vs. 48.1%; *p* = 0.01).

## Discussion

This multicenter report presents the largest analysis of long-term outcomes of allogeneic HSCT in a range of severe ADs, including several patients with increasingly characterized monogenic diseases associated with autoimmunity and immunodeficiency (40, 41).

At the last follow-up, in this heterogeneous group of patients with severe and refractory ADs, long-term complete clinical response was obtained in 67.2% of patients. Response was independent of the underlying disease (hematological vs. non-hematological AD). These data required further exploration, possibly within a prospective study.

The majority of patients underwent matched related or unrelated donor HSCT, which is generally considered the lowest risk allogeneic transplant strategy with best outcomes. Overall, NRM was 20.5% at 5-years, but this had plateaued by 2 years post-transplant. These results in patients with refractory and heavily pre-treated diseases (with some patients having already received a previous autologous HSCT) are in keeping with outcomes of allogeneic HSCT in other hematological diseases, and supportive of further exploration for selected patients with high risk ADs with a poor prognosis despite other immunomodulatory treatments including biological therapies and autologous HSCT. Potential candidates could be patients suffering a potentially life-threatening ADs with existing risk factors for an unfavorable outcome from their AD combined with low-risk factors for NRM [younger age, identical sibling or well-matched unrelated donor and low hematopoietic cell transplantation-specific comorbidity index (42)], and, in particular, in ADs with strong genetic predisposition.

Over the several decades, outcomes of allogeneic HSCT across all indications have improved due to a variety of factors, including better patient selection, donor-recipient matching and transplant techniques (1, 2, 13, 15, 16). Likewise, multivariate analysis confirmed chronological improvement in outcomes in our AD study population, especially in younger patients, supporting a “learning curve.” However, in our analysis the intensity of the conditioning regimen had no substantial association with outcomes, and, similarly, we failed to document a strong influence of donor type (between matched and mismatched donors). Nevertheless, the heterogeneity and small numbers of the study population limited the power of these comparisons. This area warrants further exploration given the potential impact of transplant technique and donor-recipient matching on the incidence of short and long-term complications.

In particular, the incidence and type of “late effects” after allogeneic HSCT in this setting is a novel feature of this analysis. Preparative regimens were relatively well-tolerated with limited regimen-related toxicity. Although there was no coordinated prospective comprehensive long-term follow-up within this group of patients, late complications were identified through a detailed questionnaire designed for this retrospective study. The spectrum of complications included 33 viral reactivations and one case of secondary malignancy. We also observed seven cases of a distinct secondary autoimmune disease, which is likely to be above the level reported follow HSCT in other contexts (43–46). A prospective study is necessary to identify the potential risk factors for the development of post-transplant AD, including details about donor chimerism and concomitant GvHD.

GvHD is also an important consideration in non-malignant indications, such as ADs. Reduction of GvHD, and potentially a mitigation of the graft-vs.-tumor (GvT) effect, is often associated with an increase in relapse in patients with hematologic malignancies; however, the occurrence of GvHD is considered an unacceptable toxicity in ADs as a GvT effect is not necessary in non-malignant diseases [9, 17. 31]. We reported an incidence of grades II-IV acute GvHD of 20.8% at 100-days and cumulative incidence of chronic GvHD of 27.8% at 5-year. By promoting bidirectional transplantation tolerance, PT-Cy strategy or alternative graft manipulation strategies (i.e., αβ T cell-depleted grafts) could further improve GvHD rates and broaden the therapeutic possibilities also in patients with ADs (8, 14, 47, 48).

Moreover, animal models at least suggest that remission of experimental AD can be achieved after non-myeloablative conditioning with mixed chimerism (43, 49, 50). Unfortunately, we did not have detailed data about the chimerism status and could not clearly prove the hypothesis that remission is independent of a complete engraftment of the donor immune system. However, with RIC regimens, which generally lead to a state of mixed chimerism in early post HSCT phase, PFS, and relapse incidence were comparable to MAC regimens.

Allogeneic HSCT combines high-dose cytotoxic therapy, donor-vs.-host alloreactivity together with replacement of a dysfunctional immune system by regenerating healthy donor immune system, with potential tolerance to allo- and auto-antigens. It is also reasonable to hypothesize that a subclinical graft-vs.-host reaction might help to eradication autoreactive B and T cells surviving the conditioning regimen, through the so-called graft-vs.-autoimmunity (GvA) effect, which has been observed in animal models (23). Unfortunately, more detailed immunological data, including donor chimerism and post-transplant immune-reconstitution, were not available in this study. In the future, fostering prospective biobanking (51) of ADs patients undergoing HSCT could enable detailed immunological analysis to further elucidate this hypothesis.

When interpreting the results of our analyses, we recognize that there are limitations such as its retrospective nature and the limited number of patients, together with the lack of uniformity in transplant protocols and follow-up process. These findings support the need to conduct prospective clinical trials to further investigate the role of allogeneic HSCT for severe treatment refractory or otherwise poor-risk ADs.

The limits of our study prevent us from addressing a definitive role for this therapeutic strategy as compared to alternative options. However, these results of this EBMT survey suggests the potential of allogeneic HSCT to achieve long-term disease remission in a large proportion of refractory ADs, with predictable toxicities, especially in younger patients, with improved outcomes in recent years.

## Data Availability

The datasets generated for this study are available on request to the corresponding author.

## Ethics Statement

This study was approved and supported by the Autoimmune Diseases Working Party (ADWP), Inborn Errors Working Party (IEWP), and Pediatric Working Party (PDWP) of the European Society for Blood and Marrow Transplantation (EBMT).

## Author Contributions

RG, JS, DF, FC, MBa, MA, TA, AG, and PB designed the study. RG, MBa, ML, FC, and JS performed analysis and interpretation of data. JS, MA, DF, FC, AG, and PB provided scientific advice and supervision. RG, JS, FC, MBa, and ML wrote the article. The EBMT provided resources via the working parties, data office, and registry. All authors have approved the final version of the manuscript and contributed to patient clinical care and data collection.

### Conflict of Interest Statement

JS declares honoraria for speaking by Sanofi, Jazz, Janssen, Mallinckrodt, Actelion and Gilead, and IDMC membership for Kiadis Pharma. PB declares research grants from Neovii, Riemser, Medac, advisory board from Novartis, Cellgene, Amgen, Medac, Servier (Institut.), patent and royalties from Medac. The remaining authors declare that the research was conducted in the absence of any commercial or financial relationships that could be construed as a potential conflict of interest. The handling editor declared a shared affiliation, though no other collaboration, with one of the authors TA.
